# A biomechanical study comparing the compression force and osseous area of contact of two screws fixation techniques used in ankle joint arthrodesis model

**DOI:** 10.1186/s13018-024-04906-6

**Published:** 2024-08-10

**Authors:** Annabelle Weigert, Manuel Kistler, Leandra Bauer, Adrian C. Kussmaul, Alexander M. Keppler, Boris Michael Holzapfel, Bernd Wegener

**Affiliations:** 1 Department of Orthopedics and Trauma Surgery, Musculoskeletal University Center Munich (MUM), University Hospital, LMU Munich, Munich, Germany; 2 Department of Experimental Orthopedics, University Hospital Jena, Campus Eisenberg, Waldkliniken Eisenberg, Eisenberg, Germany

**Keywords:** IOFix, Headless compression screws, Area of contact, Arthrodesis ankle joint, Compression force, Biomechanics

## Abstract

**Introduction:**

Arthrodesis of a (diseased) ankle joint is usually performed to achieve pain relief and stability. One basic principle of arthrodesis techniques includes rigid fixation of the surfaces until union. It seems plausible that stable anchoring and homogeneous pressure distribution should be advantageous, however, it has not been investigated yet. The aim is to achieve uniform compression, as this is expected to produce favorable results for the bony fusion of the intended arthrodesis. Numerous implants with different biomechanical concepts can be used for ankle fusion. In this study, headless compression screws (HCS, DePuy Synthes, Zuchwil, Switzerland) were compared biomechanically to an alternative fixation System, the IOFix device (Extremity Medical, Parsippany, NJ, USA) in regard to the distribution of the compression force (area of contact) and peak compression in a sawbone arthrodesis-model (Sawbones® Pacific Research Laboratories, Vashon, WA, USA).

This study aims to quantify the area of contact between the bone interface that can be obtained using headless compression screws compared to the IOFix. In current literature, it is assumed, that a large contact surface with sufficient pressure between the bones brings good clinical results. However, there are no clinical or biomechanical studies, that describe the optimal compression pressure for an arthrodesis.

**Material and methods:**

Two standardized sawbone blocks were placed above each other in a custom-made jig. IOFix and headless compression screws were inserted pairwise parallel to each other using a template for a uniform drilling pattern. All screws were inserted with a predefined torque of 0.5 Nm. Pressure transducers positioned between the two sawbone blocks were compressed for the measurement of peak compression force, compression distribution, and area of contact.

**Results:**

With the IOFix, the compression force was distributed over significantly larger areas compared to the contact area of the HCS screws, resulting in a more homogenous contact area over the entire arthrodesis surface. Maximum compression force showed no significant difference.

**Conclusion:**

The IOFix system distributes the compression pressure over a much larger area, resulting in more evenly spread compression at the surface. Clinical studies must show whether this leads to a lower pseudarthrosis rate.

## Introduction

Arthrodesis of the ankle or foot joints (i.e. triple arthrodesis, transverse tarsal joint arthrodesis) is commonly performed in the operative treatment of symptomatic, end-stage osteoarthritis and/or acquired flatfoot deformity. These conditions may be posttraumatic/degenerative, malformation- or infect-related. The fusion of a diseased joint is performed to achieve pain relief and stability. One frequent complication following ankle arthrodesis is non-union/pseudarthrosis. In current literature, variable union rates in ankle arthrodesis following open or arthroscopic approaches with different fixation techniques have been reported. Nonunion rates of ankle arthrodesis range from 2 to 47% [1–5]. Non-union of the ankle arthrodesis can be caused by various factors. These include patient-specific problems, such as smoking, arteriosclerosis, or diabetes, and can not be influenced by the surgeon. Nevertheless, factors that can be influenced by the surgeon for good and satisfactory clinical results include the surgical technique to perform an arthrodesis and the selection of an adequate implant to achieve compression. Another factor that could be of importance in the occurrence of pseudarthrosis is the failure to obtain and maintain compression across the fusion during the healing process due to implant failure. [6, 7]

One basic principle of arthrodesis includes, among others, rigid fixation of the surfaces until union, which can be achieved using variable fixation devices with different biomechanical properties [8, 9]. Numerous biomechanical studies investigated the stiffness of different implants used for ankle fusion [10–13]. In these studies, an arthrodesis was performed or simulated in either sawbone models or cadaver specimens with different implant constructs (double plating versus screws; external fixator versus crossed-screw; compressed external fixator versus an uncompressed interlocking nail versus a compressed interlocking nail versus two different three-screw techniques; External ring fixation versus screw fixation) and were biomechanically compared. Using material testing machines, their focus was on investigating the load to failure (N), stiffness of the constructs (N/mm), the ultimate bend, torque, and compression as well as quantifying the bending and torsional stiffness of each arthrodesed joint.

Additionally, some biomechanical studies focused on screw design and their mechanical performance or their pullout strength [1, 14, 15]. In other biomechanical studies, the static performance of different implants and their properties (thread length, cannulation, etc.) were investigated. The overall goal was to determine how screw thread geometry, tapping, and cannulation affect the holding power of screws in cancellous bone and determine whether current designs achieve maximum holding strength.

Burchard et al. and Roth et al. investigated intramedullary fixation in arthrodesis of the 1st metatarsophalangeal joint. They aimed to analyze the load to failure, cycles to failure, and stiffness compared to traditional fusion methods, such as plates and screws [16, 17]. However, the optimal compression force in an arthrodesis was not investigated.

Due to the design of different screws, the compression force can reach variable peaks during implantation, but more importantly, they may spread the compression more uniformly and thus produce a larger contact area at the arthrodesis site of the ankle joint [18]. Implant design has an influence on mechanical properties e.g. compression force [19].

So far, there are no biomechanical studies that describe the optimal pressure for arthrodesis or osteosynthesis. Insufficient contact surface and lack of compression can be the cause of pseudarthrosis [20, 21]. It is therefore important to investigate implants with different properties regarding their ability to achieve compression and contact surface. It is assumed that a higher compression force at the arthrodesis site and greater distribution of this force (contact area) could have a positive effect on the outcome and will therefore be investigated in this study.

## Material and methods

### Implants and test set up

**Fig. 1:**
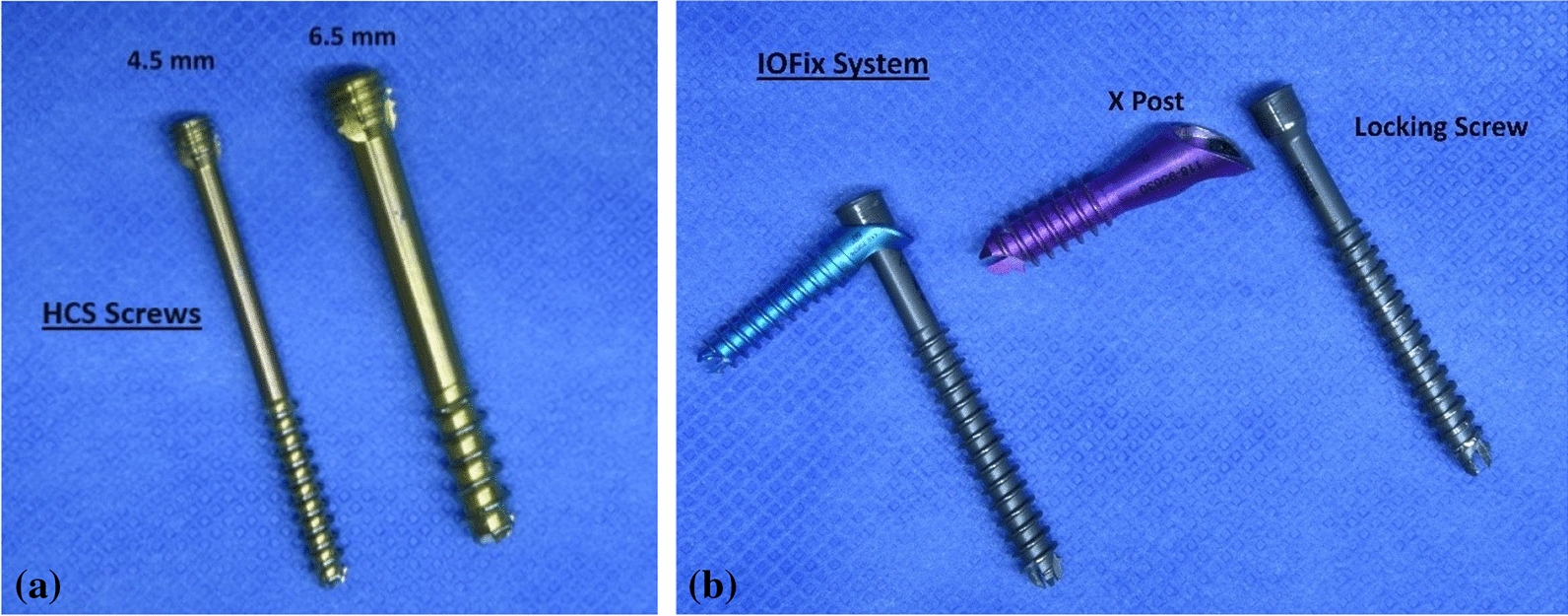
**a** and **b** HCS screws and IOFix system. **a** HCS screws with a diameter of 4.5 mm and 6.5 mm are shown. **b** The IOFix small (blue) and IOFix medium (magenta) are shown. The IOFix system consists of 2 screws: the X Post and locking screw. The eyelet on the X-Post’s head is prefabricated for the locking screw to get engaged at a 60° angulation. The exact screw parameters for the HCS and IOFix Systems small/medium/large are also shown in Table 1

**Fig. 2:**
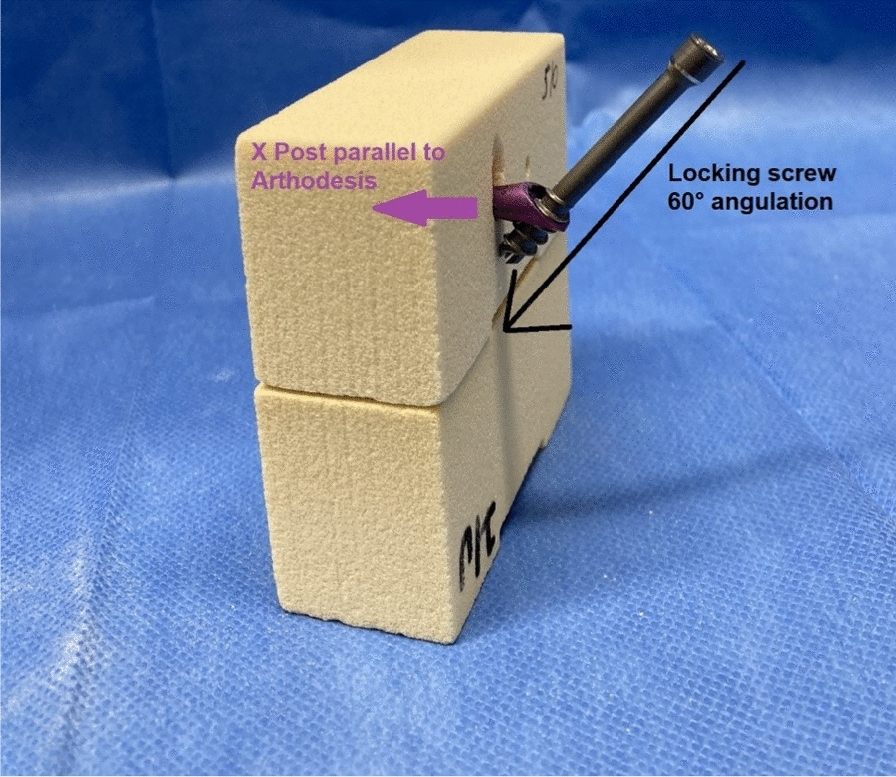
Insertion of the IOFix system. The insertion of the IOFix system into the sawbone model is shown: First, the X Post (magenta) is inserted parallel to the arthrodesis plane, after predrilling a pilot hole with the cannulated drilling bits. Then, the locking screw is inserted in the eyelet of the X-post’s head, and is then passed across the arthrodesis site with 60° angulation

For testing, two types of screws, which are commonly used for fixation of arthrodesis of the ankle joint, with different compression principles, were selected for this biomechanical analysis. Included were Headless Compression Screws (HCS, DePuy Synthes, Zuchwil, Switzerland) with two different diameters (4.5 mm and 6.5 mm), and the IOFix system in three sizes (Extremity medical, Parsippany, NJ, USA). Both screw-types are shown in Fig. 1. The IOFix system is a fixed-angle device, comprising two screws. The first screw called the X-post, is inserted parallel to the arthrodesis plane. The second screw is a locking screw, which is inserted through the prefabricated eyelet of the X-post’s head at a 60° angle, shown in Fig. 2.

In total, 5 groups were biomechanically tested and compared to each other: (1) 4.5 mm HCS screws, (2) 6.5 mm HCS screws, (3) IOFix small with the diameters X-Post 6.6 mm and locking screw 4.0 mm, (4) IOFix medium with diameters X-Post 8.0 mm and locking screw 5.0 mm, and (5) IOFix large with diameters X-Post 9.5 mm and locking screw 6.5 mm. The description of the HCS and IOFix screws is shown in Table 1a and b.

**Table 1:** Description of the HCS and IOFix screws

Group	Pilot hole	Length (L), thread length (TL), thread pitch increments (TP)
(a) Description of the Headless Compression screws		
HCS 4.5 mm	3.2 mm	L 50 mm, TL 20 mm, TP 2 mm increments
HCS 6.5 mm	5.0 mm	L 50 mm, TL 16 mm, TP 5 mm increments

Group	X post: diameter, pilot hole, length	Locking screw: Diameter (D), length (L), thread length (TL), thread pitch increments (TP)
(b) Description of the IOFix screws		
IOFix small	6.6 mm, 3.4 mm, 30 mm	D 4.0 mm, L 50 mm, TL 15 mm, TP 5 mm increments
IOFix medium	8.0 mm, 4.5 mm, 30 mm	D 5.0 mm, L 50 mm, TL 15 mm, TP 5 mm increments
IOFix large	9.5 mm, 4.5 mm, 30 mm	D 6.5 mm, L 50 mm, TL 16 mm, TP 5 mm increments

In Table 1a and Table 1b the biomechanical parameters of the HCS screws and IOFix screws are shown

**Fig. 3:**
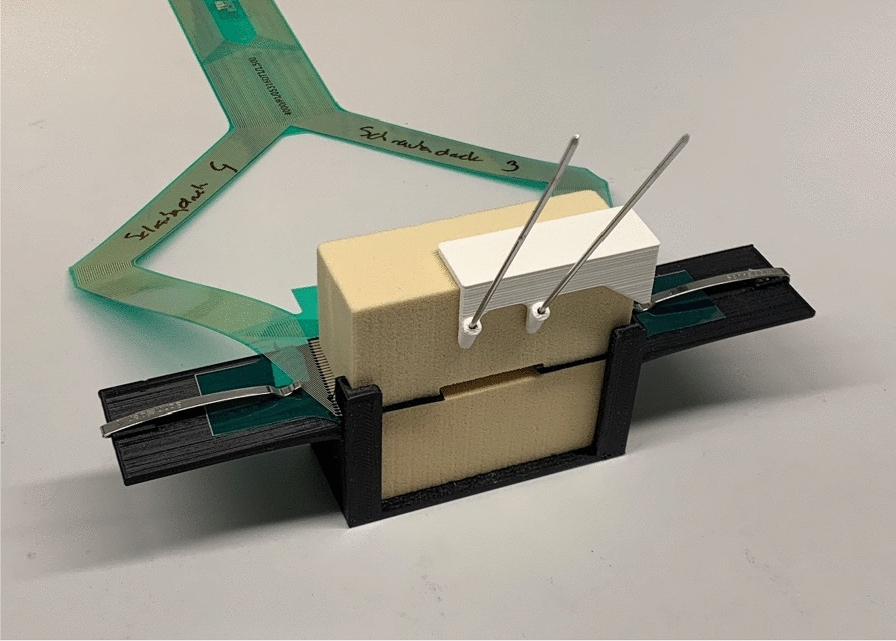
The experimental set up. In a custom made jig, two saw bone blocks are stacked on each other, imitating an arthrodesis model. The template is placed on top for standardized insertion of the K-wires/screws (standardized height from the arthrodesis gap and distance between the screws). Two pressure sensor transducers (green foils) are placed between the saw blocks

For the experimental setup shown in Fig. 3—which is based on the set up by Mueller et al. [22]—two stacked sawbone blocks (Sawbones® Pacific Research Laboratories, Vashon, WA, USA) were stabilized in a custom-made container to prevent the blocks from tilting. Two pressure sensor transducers (K-Scan 4000, Tekscan, Inc., Boston, MA, USA) were placed between the sawbone blocks and held in place by the clamps attached to each side of the container, to prevent the sensors from slipping.

A template was positioned on the upper sawbone to ensure a standardized uniform height and distance between the screws to be inserted. The K-wires were inserted through the prefabricated holes of the template (with a defined height of 20 mm to the arthrodesis plane and 20 mm distance between the screws) in the template. After removing the template, the screw holes were pre-drilled by over-drilling the guidewires with cannulated drill bits. According to the manufacturer’s instructions, the pilot hole for the 4.5 mm HCS screws was drilled using a 3.2 mm cannulated drilling bit and a 5.0 mm cannulated drilling bit for the 6.5 mm HCS screws.

For the IOFix system, the pilot hole for the X-post is predrilled—according to the manufacturer’s manual—with 2.0 mm/3.4 mm/4.5 mm cannulated drill bits, respectively for IOFix small/medium/large. After insertion of the X-post parallel to the arthrodesis plane, the locking screw is inserted in the eyelet of the X-post’s head and is then passed across the arthrodesis site with a 60° angulation. The lag screw gets engaged in the eyelet of the X-post, resulting in a more uniform compression across the fusion site. [18] Three different sizes of the IOFix device were tested—IOFix small, medium, and large (Table 1).

For the respective group, two identical HCS screws and two identical IOFix devices were inserted pairwise parallel to each other in a straight pattern. A predefined torque was applied to all screws (0.5 Nm) simultaneously to their insertion, which was detected by using a digital screwdriver with an integrated digital sensor (PCE-TM 80, PCE GmbH, Meschede, Germany). Rotational stability was given for all five groups.

**Fig. 4:**
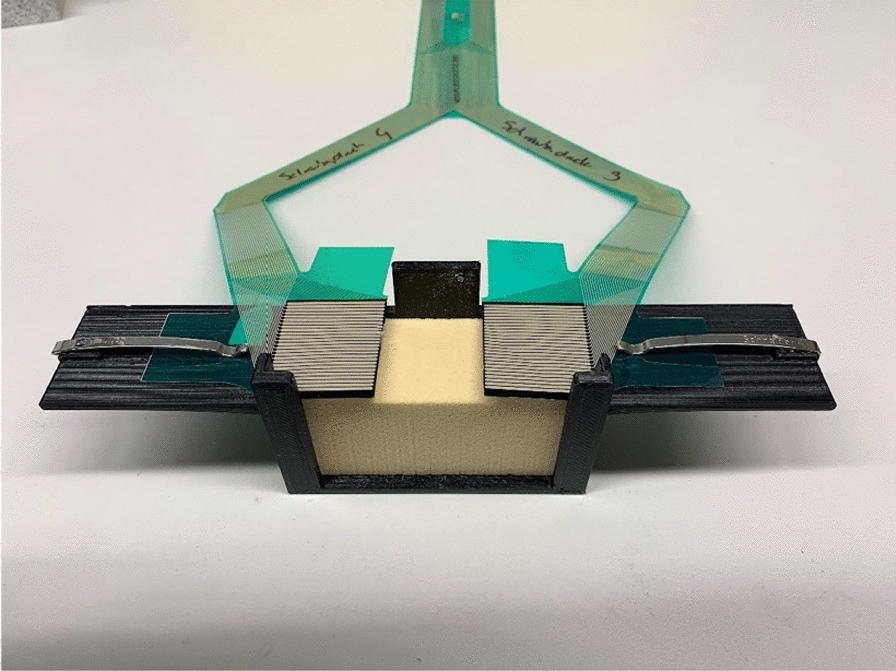
Flexiforce pressure sensor transducer. Two flexiforce pressure sensor transducers connected to a measuring device were placed between the sawbone blocks and held in place by the clamps on each side of the container to prevent the sensors from slipping. Two parameters can be determined from the flexiforce pressure transducer foils: Peak compression (on every single pixel) and how many pixels around the inserted screw are activated (area of contact)

Seven measurements per group (n = 35) were performed on pre-assembled saw bone blocks with a density of 15 pounds per cubic foot (solid foam; personalized blocks). The compression force at the arthrodesis site was measured and recorded using two flexiforce pressure transducers, which were connected to a measuring device (Fig. 4). The pressure sensors were calibrated to display compression force and the contact area—which is created when the blocks are pressed together—in Megapascal (MPa) and in square millimeters (mm^2^).

**Fig. 5:**
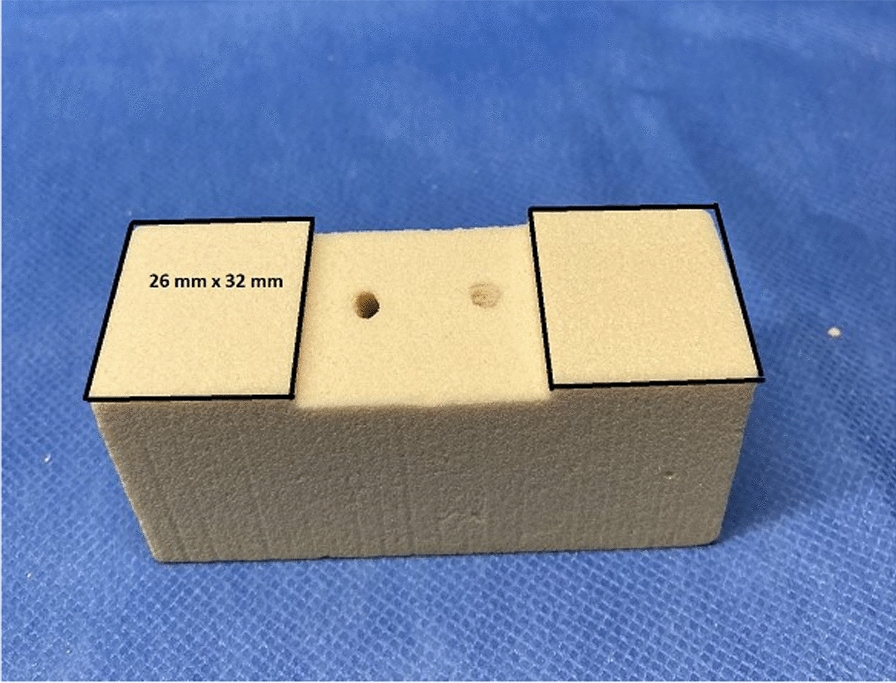
Area of contact at the arthrodesis gap. On the sawbone block, each (articulating) surface has a surface area of 832 mm^2^ (26 × 32 mm), which is covered by the flexiforce pressure sensors. The maximum area of pixels that can be activated on the flexiforce pressure sensor foils is 832 mm^2^ on the right and left side

Each (articulating) surface of the sawbone block has a surface area of 832 mm^2^ (26 × 32 mm), as seen in Fig. 5. Because in this study an arthrodesis is imitated, lamination of the sawbone blocks to simulate the base plate was omitted.

A calibration was carried out at the beginning of each series of measurements. After final tightening of the screws, the pressure transducer was initiated to start the digital recording of the compression force and measurement of the force distribution (area of contact). This was achieved by the pressure-sensitive films being positioned within the arthrodesis and, therefore, active to determine the average contact area over a time span of 10 s.

### Data analysis and statistics

A post hoc power analysis using G*Power (version 3.1.9.7; Franz Paul, Kiel, Germany) was performed to determine the power of the study. Based on the results of the F-test and ANOVA, an effect size of 0.90 was calculated. With this effect size, an α of 0.05, and the sample size of 35, a power of 0.98 was calculated.

For statistical analysis, all further data processing was performed with MATLAB (MathWorks Inc., Natick, MA, USA). Peak pressure was calculated by averaging the values which were measured in a time span of 10 s over a window with the eight surrounding values, to avoid artifacts (according to [23]). The contact area reflected the number of pixels that had a pressure value greater than 0 MPa.

Quantitative parameters are given as mean, median, and standard deviation, and were then calculated for the seven sawbones per screw-pair. Statistical analysis was performed using SPSS (IBM SPSS Statistics 27). In order to compare the HCS 4.5 mm/HCS 6.5 mm groups with the IOFix small/medium/large groups, a test for variance homogeneity was calculated first, followed by an analysis by means of one-factor analysis of variance (ANOVA). The results were presented with boxplots. The significance level of *p* < 0.05 was accepted as a statistically significant difference.

## Results

### Compression force

With the predefined torque of 0.5 Nm for every inserted screw, there is no significant difference in the maximum generated compression force (peak compression), when comparing the HCS 4.5 mm, HCS 6.5 mm, IOFix small and IOFix medium.

However, when comparing the peak compression force that was generated by the IOFix large device, compared to either HCS 4.5 mm or HCS 6.5 mm screws, significant differences were detected: While the HCS 4.5 mm screws generated 0.22 MPa and the HCS 6.5 mm 0.41 MPa of compression force, the IOFix large device produced a compression force of 0.84 MPa, being significantly larger than the compression obtained by HCS 4.5/6.5 mm (*p* < 0.001, *p* < 0.012). The results are shown in Table 2.

**Table 2:** Mean Peak of Pressure in [MPa]

Screw	Mean ± Standard deviation	*p*-value	
		HCS 4,5 mm	HCS 6,5 mm
HCS 4,5mm	0.22 (± 0.17)	-	n.s
HCS 6,5mm	0.41 (± 0.21)	n.s	-
IOFix small	0.36 (± 0.09)	n.s	n.s
IOFix medium	0.41 (± 0.25)	n.s	n.s
IOFix large	0.84 (± 0.09)	< 0.001	< 0.012

Statistical analysis of the mean peak pressure is demonstrated: It shows that, there is no significant difference between the peak pressures produced by the HCS 4.5 mm/6.5 mm screws and the IOFix small and medium. However, there is a significant difference in mean peak pressures when IOFix large is implanted (*p* < 0.001, *p* < 0.012), compared to mean peak pressures of HCS 4.5/6.5 mm

### Distribution of the force (Area of contact)

**Fig. 6:**
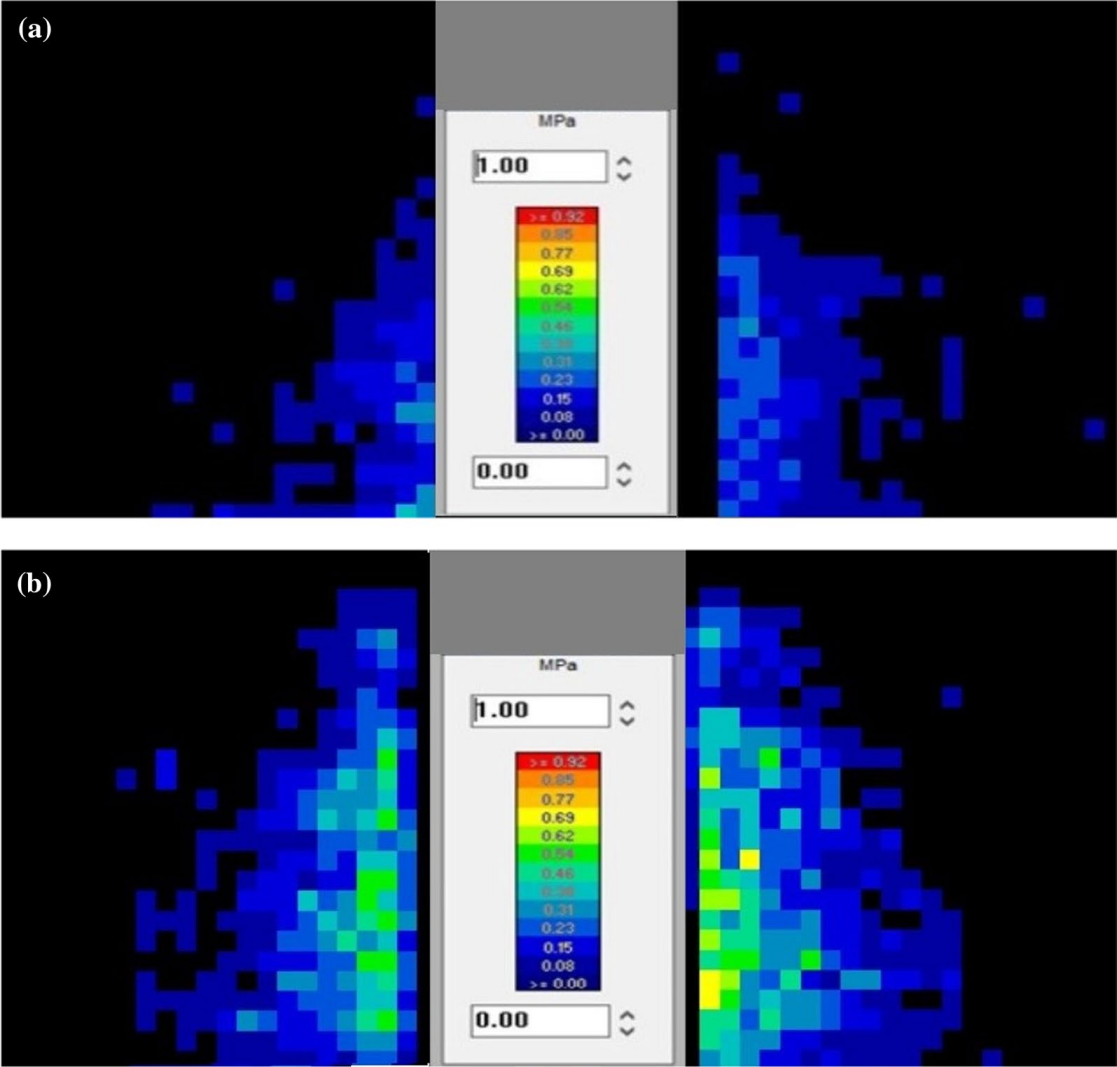
**a** Activated Pixels produced by 4.5 mm HCS screws. This figure visualizes two parameters: First, it shows how many pixels of the pressure sensor foils are activated (area of contact). Second, how high the pressure on one single pixel is (the Color scale encodes: navy blue 0.08 MPa vs. red 0.92 MPa). In the group of HCS 4.5 mm screws, the area with activated pixels (contact area between the saw bone blocks) measured 159.5 mm^2^, which made up 19% of the surface. **b** Activated Pixels produced by IOFix small.This figure visualizes two parameters: First, it shows how many pixels of the pressure sensor foils are activated (area of contact) by the IOFix small system. Second, it shows how high the pressure on one single pixel is (Color scale: blue 0.08 MPa, green 0.54 MPa, yellow 0.69 MPa). IOFix small activate pixels of the pressure sensor foils with an area of 420.6 mm^2^, making up 50% of the total surface. Also, the individual activated pixels show higher pressure, as they are blue, green and yellow color coded

In the group of HCS 4.5 mm screws, the mean contact area between the saw bone blocks measured 159.5 mm^2^, which made up 19% of the surface area (Fig. 6a). In the group of HCS 6.5 mm, the mean contact area increased to 171.4 mm^2^ (20% of the max. surface area). Testing the IOFix small, there is a disproportionately large increase of the contact area, compared to the contact area produced by 6.5 mm HCS screws—an area of 420.6 mm^2^ of the pressure sensor pixels were activated, which makes up 50% of the surface (Fig. 6b).

Comparing HCS 4.5/6.5 mm screws to IOFix small/medium or large, significant differences could be detected (*p* = 0.012, *p* = 0.022; *p* = 0.022, *p* = 0.037; *p* =  < 0.001, < 0.011, respectively for HCS 4.5 mm and HCS 6.5 mm), shown in Table 3.

**Table 3:** Contact Area [mm^2^]

Screw	Mean ± Standard deviation	*p*-value	
		HCS 4,5 mm	HCS 6,5 mm
HCS 4,5mm	159.46 (± 111.65)	–	n.s
HCS 6,5mm	171.41 (± 78.16)	n.s	–
IOFix small	420.57 (± 173.50)	0.012	0.022
IOFix medium	403.21 (± 194.81)	0.022	0.037
IOFix large	660.58 (± 108.19)	< 0.001	< 0.011

The number of activated pixels (mm^2^) of the pressure sensor foils are shown: A comparison of HCS screws with IOFix screws shows that there is a statistical significance between HCS 4.5 mm to IOFix small/medium/large and HCS 6.5 mm to IOFix small/medium/large. Therefore, the area of contact is greater when implanting IOFix systems, compared to HCS 4.5/6.5 mm screws

## Discussion

This study evaluated the influence of different implants on the compression force and area of contact in a sawbone-based ankle-arthrodesis model. Regarding the contact area, the main finding of this study was that the IOFix system spreads the compression force more evenly over the surface, creating a significantly larger contact area between the sawbones at the arthrodesis site than two parallel inserted HCS screws, which is visualized in Figs. 6a and b. The mechanism of IOFix is the additional implantation of the X-Post prior to inserting the angular stable screw, which distributes compression forces across a greater surface area. One further finding of this study was, that the IOFix large also achieved higher values for the compression pressure than HCS screws did.

From these flexiforce pressure transducer foils, two important parameters can be determined. The first parameter indicates how high the maximum built-up compression on the specific single pixel is (peak compression). The second parameter displays how many pixels surrounding the inserted screw are activated (area of contact). There is no scientific parameter that represents the biomechanic situation at the arthrodesis gap better than the activated pixels, i.e. the area of contact.

Since compression of only a small area of the overall surface or also too much compression on this partial surface are risk factors for the development of pseudarthrosis, an ideal arthrodesis should have moderate compression with an even distribution across the matched bone surfaces, to minimize stress at areas of high peak contact, as well as neutralize shear and bending forces. By avoiding uneven compression across imperfectly matched surfaces, areas of high peak contact stress are minimized and could reduce the risk of bone resorption by osteoclasis, failure of fixation, and non-union [8].

Compared to the IOFix screws, which achieved a larger and more uniform pressure distribution across the arthrodesis, 4.5 mm HCS, and 6.5 mm HCS screws only showed localized compression by activating pixels of the pressure sensors around the screw insertion point. The HCS screws tended to concentrate a high peak pressure nearest where they were inserted. This stress may affect bone resorption. Bone resorption in areas of high peak contact stress within an arthrodesis may lead to progressive loss of bone interdigitation, gapping, and non-union at the interface [24]. It is therefore important to know the biomechanical properties of the implants used.

In current literature, there is a total of nine studies that investigate the clinical outcome and biomechanical properties of the IOFix device on foot and ankle joints [16, 17, 25–31]. Seven of these publications report on the fusion of the 1st metatarsophalangeal joint, one case series reports on the fusion of the talonavicular joint, while there is only one biomechanical study performed on cadaveric ankle joints by Parker et al.[18, 31]

Parker et al. [18] had similar findings to those in this study, though, in their study, they compared the IOFix to one single 6.5 mm, partially-threaded, cannulated cancellous lag-screw with a washer in cadaveric ankle joints. However, it needs to be considered that a cadaver study may not necessarily supply reproducible results. In addition, it would not be recommendable to use only one single screw in an ankle-arthrodesis, as a large area has to be treated and there is no rotational stability given. Furthermore, it also remains unclear whether there are (statistically significant) differences between the IOFix devices, if the devices have different sizes (like small, medium, and large). This study provides better comparable results with the variables being kept the same and standardized because individual differences like bone density, age, gender, etc. which are typical for cadaver studies are not being accounted for.

Furthermore, in this study, three different sizes of the IOFix screws (IOFix small/medium/large) and two different sizes of the HCS screws (HCS 4.5 mm and 6.5 mm) were tested and compared to each other, using artificial saw bone blocks, each with the same density and the same size of possible contact area. A template for drilling to guarantee a standardized test set-up was used. Due to the test setup and the comparison of different sizes/diameters of each screw type, the results can also be transferred to smaller or larger joints.

Using sawbones with the same density could be a potential advantage, as the variable, age-dependent bone density of cadavers did not have to be taken into account for evaluation. Also, this may be relevant for the possible influence the bone quality might have on screw stripping. In optimal osseous settings, the differences of the large IOFix screws may be significant in a sawbone model, however, measurements might not be advantageous in the clinical setting. As mentioned before, bone quality must be taken into account in the surgeon’s preoperative planning, e.g. cystic lesions should be filled when in doubt. Another important difference to a cadaver-based study is that the articulating saw-bone planes were flat and perfectly aligned to each other, limiting the risk of an uneven arthrodesis plane/malalignment. This test setup is an idealized arthrodesis model, which can easily be recreated and the results are reproducible. With this test setup, the biomechanical properties of different screws can be tested and compared to each other.

Nevertheless, this study has several limitations. As with all biomechanical studies, it has the inherent problem that only time-zero compression force can be evaluated, which represents the intraoperative situation. The initial period of clinical healing, though, is the most important and this was measured in this study. Many other factors are also important, especially in the early stages of clinical healing, such as insufficient compression or inadequate immobilization [22].

What is important for reproducible biomechanical results, is at the same time a limitation of this study. It is a sawbone test setup, not resembling anatomical variation. This construct was not subject to physiological loading, including compression and shear.

Furthermore, the size of the IOFix large screws could be relevant- in case of a revision surgery due to lack of bony healing, less bone stock is present*.* Finally, the drilling and insertion of the screws were standardized in the laboratory, not accounting for any technical and visual problems that might occur during drilling in the operating room. However, the aim of this study was to compare different implants used for ankle arthrodesis in terms of their ability to create a contact surface between the bones and that the test setup can provide reproducible results.

## Conclusion

The aim of this biomechanical study was to demonstrate that the tightening of various implants builds up different levels of compression and may activate a larger or smaller area of the pressure sensor, which is positioned in an arthrodesis gap. This study demonstrated that the distribution of forces and the activated area surrounding the arthrodesis screw are dependent on the implant used: two parallel inserted IOFix systems distribute the compressive forces across a significantly greater surface area, leading to an optimized distribution of compression forces at the arthrodesis-site. Also, they show a larger area in force distribution compared to two parallel HCS screws. This could offer clinical advantages. Clinical studies must show whether the use of specific implants can reduce the rate of pseudarthrosis.

## Data Availability

The data presented in this study are available on request from the corresponding author.
